# ISKNV Triggers AMPK/mTOR-Mediated Autophagy Signaling through Oxidative Stress, Inducing Antioxidant Enzyme Expression and Enhancing Viral Replication in GF-1 Cells

**DOI:** 10.3390/v16060914

**Published:** 2024-06-04

**Authors:** Tsai-Ching Hsueh, Pin-Han Chen, Jiann-Ruey Hong

**Affiliations:** 1 Laboratory of Molecular Virology and Biotechnology, Institute of Biotechnology, National Cheng Kung University, Tainan 701, Taiwan; 2 Department of Biotechnology and Bioindustry Sciences, National Cheng Kung University, Tainan 701, Taiwan

**Keywords:** ISKNV, DNA virus, autophagy, mTOR, ROS, oxidative stress, antioxidant NAC, fish

## Abstract

Infectious spleen and kidney necrosis virus (ISKNV) infections can induce the process of host cellular autophagy but have rarely been identified within the molecular autophagy signaling pathway. In the present study, we demonstrated that ISKNV induces ROS-mediated oxidative stress signals for the induction of 5′AMP-activated protein kinase/mechanistic target of rapamycin kinase (AMPK/mTOR)-mediated autophagy and upregulation of host antioxidant enzymes in fish GF-1 cells. We also examined ISKNV-induced oxidative stress, finding that reactive oxidative species (ROS) increased by 1.5-fold and 2.5-fold from day 2 to day 3, respectively, as assessed by the H_2_DCFDA assay for tracing hydrogen peroxide (H_2_O_2_), which was blocked by NAC treatment in fish GF-1 cells. Furthermore, ISKNV infection was shown to trigger oxidative stress/Nrf2 signaling from day 1 to day 3; this event was then correlated with the upregulation of antioxidant enzymes such as Cu/ZnSOD and MnSOD and was blocked by the antioxidant NAC. Using an MDC assay, TEM analysis and autophagy marker LC3-II/I ratio, we found that ROS stress can regulate autophagosome formation within the induction of autophagy, which was inhibited by NAC treatment in GF-1 cells. Through signal analysis, we found that AMPK/mTOR flux was modulated through inhibition of mTOR and activation of AMPK, indicating phosphorylation levels of mTOR Ser 2448 and AMPK Thr 172 from day 1 to day 3; however, this process was reversed by NAC treatment, which also caused a reduction in virus titer (TCID_50%_) of up to 1000 times by day 3 in GF-1 cells. Thus, ISKNV-induced oxidative stress signaling is blocked by antioxidant NAC, which can also either suppress mTOR/AMPK autophagic signals or reduce viral replication. These findings may provide the basis for the creation of DNA control and treatment strategies.

## 1. Introduction

Iridovirus has been shown to cause serious damage to the global population, being responsible for significant losses of up to 20 species within the economic aquaculture industry [1,2]. The large dsDNA virus family of iridovirus includes five genera: *Chloriridovirus*, *Ranavirus*, *Iridovirus*, *Lymphocystivirus*, and *Megalocytivirus* [3]. Particles of iridovirus are 120–200 nm in diameter and exhibit icosahedral symmetry; they contain up to 120 open reading frames (ORFs) [4]. New and emerging strains of megalocytiviruses may infect a wide range of freshwater fish and tropical marine fish. In particular, the ISKNV strain affects species including sea bass, red sea bream, groupers, gourami, cichlids, angel fish and lamp eyes; this viral strain causes very similar diseases in each species and subsequently causes significant mortality in populations of these economically important fish [1,2,5]. Thus, looking at the mechanisms of the molecular pathogenesis of ISKNV infection, we must develop new and effective prevention strategies that are able to block the virus from progressing from its nascent stages. 

Oxidative stress occurs in cells when the production of ROS exceeds the cell’s endogenous antioxidant defenses [6]; it may correlate with Nrf2 stress transcription factor activation [7,8]. The major cellular agents of defense against ROS include superoxide dismutases (SODs) and catalase [9,10], metabolites that counter the overproduction of ROS. SODs catalyze the dismutation of superoxide anion (O_2_^•−^) to hydrogen peroxide (H_2_O_2_) and molecular oxygen (O_2_) and are located in the cytoplasm (Cu/ZnSOD; SOD1) and mitochondria (MnSOD; SOD2) [11]. Catalase is a tetrameric iron porphyrin protein located in the peroxisome that scavenges H_2_O_2_, thus producing H_2_O and O_2_ [12], which act to reduce cellular damage [13]. Very few studies have investigated DNA-virus-induced oxidative stress and diseases in fish. While ROS are now known to be important regulatory molecules involved in triggering signals [14], whether they can regulate viral replication remains unknown.

Autophagy is a physical function for cell defense against intracellular microbes during which the intracellular microbes are fused to lysosomes for degradation [15]. Autophagy contributes to the activation of innate antiviral immunity [16,17] and adaptive immune responses by delivering virus-derived peptides to lymphocytes for presentation by major histocompatibility complex molecules [18,19,20]. The mTOR is a protein kinase that regulates autophagy by stimulating protein synthesis and inhibiting the induction of autophagy [21]. In mammalian cells, 5′AMP-activated protein kinase (AMPK) is the main upstream regulator of mTOR. mTOR activation has recently been shown to regulate unc-51-like autophagy activating kinase 1 (ULK1) by mediating the phosphorylation of Ser757, thereby blocking the initiation of autophagy [22].

Some viruses have evolved to develop molecular mechanisms that allow them to escape from or inhibit autophagy, thereby increasing their infectivity [23]. However, recent studies have indicated that ISKNV infection can induce the generation of ROS during the early stages of replication, which triggers an oxidative stress response that is correlated with apoptotic Bax/Bak-mediated cell death; however, few studies have linked ISKNV infection with the regulation of autophagy [8]. In the present study, we investigated the molecular mechanisms of ISKNV-induced ROS/AMPK/mTOR-mediated autophagy in fish cells, including examination of signaling pathways and downstream targets. We also examined the impact of autophagy on ISKNV infection and found that it enhanced viral replication. Our findings regarding the molecular pathogenesis of ISKNV infection could help us to identify potential targets of antioxidant NAC that could be utilized for the treatment of ISKNV-induced diseases and the development of strategies for reducing the high mortality rates of DNA viruses.

## 2. Materials and Methods

### 2.1. Cell Lines and Virus

GF-1 cells from a continuous cell line derived from grouper fins of *Epinephelus coioides* were grown at 28 °C in Leibovitz’s L-15 medium (11415-114 ThermoFisher Scientific Inc., Waltham, MA, USA) supplemented with 5% fetal bovine serum and 25 μg/mL of gentamycin. Naturally infected red grouper larvae, which were collected in 2016 in the Tainan Prefecture, were the source of the ISKNV Kaohsiung No. 1 (ISKNV KN1) that was used for cell infection in this study. The virus was purified as described by Mori et al. [24] and then stored at −80 °C until use. The viral titer was determined using a TCID_50_ assay, as reported by Dobos et al. [25].

### 2.2. Quantification of Cell Viability

GF-1 cells were pretreated with 2 mM NAC for 2 h before virus infection. The ISKNV-infected GF-1 cells (MOI = 1) and uninfected cells were then incubated at 28 °C for 0, 1, 2, 3, 4 and 5 days. At the end of each culture period, the cell layers were washed with PBS and treated with 0.1% trypsin-EDTA (0.5 mL, 1–2 min; Gibco, Grand Island, NY, USA). Cell viability was determined (in triplicate) using a trypan blue dye exclusion assay [26]. Each point represents the mean viability of three independent experiments ± the standard error of the mean (SEM). Data were analyzed using either paired or unpaired Student’s *t*-tests as appropriate. A value of *p* < 0.05 was taken to represent a statistically significant difference between mean values of groups.

### 2.3. ROS (Hydrogen Peroxide, H_2_O_2_) Generation Assays

The ROS in living cells were evaluated using the Image-iT LIVE Green Reactive Oxygen Species Detection Kit (I36007, Molecular Probes, Eugene, OR, USA). This assay functions through staining using carboxy-DCF, which is a fluorogenic marker of ROS formation in live cells. Briefly, GF-1 cells (10^5^ cells/mL) were cultured to monolayer confluence for 20 h in Petri dishes of 60 mm diameter or 6-well plates, rinsed twice with PBS, pretreated with 2 mM NAC for 2 h, and then infected with ISKNV (MOI = 1) for 0, 1, 2, 3, 4, or 5 days at 28 °C. At the end of each incubation period, the culture medium was aspirated, and the cells were washed with PBS and incubated in the dark for 30 min with 500 μL of working solution (25 μM of carboxy-DCF in PBS (10 mM of PO_4_^3−^, 137 mM of NaCl and 2.7 mM of KCl; pH 7.4)). The samples were immediately examined via fluorescence microscopy using a 100 W halogen bulb for 0.5 s (excitation: 488 nm; emission: 515 nm longpass filter). The percentage of fluorescent cells (n = 200) at each time point was determined in triplicate, with each point representing the mean of three independent experiments and each error bar representing the standard error of the mean. All data were analyzed using either the paired or unpaired Student’s *t*-test, as appropriate. A *p*-value below 0.01 indicated that there was a statistically significant difference between the mean values of compared groups [27]. 

### 2.4. Labeling of Autophagic Vacuoles with Monodansylcadaverine (MDC)

Changes in the autophosome formation that occurred during ISKNV-induced autophagy were examined using MDC dye. The GF-1 cells were seeded at 1 × 10^5^ cells per mL in a 60 mm Petri dish for at least 20 h prior to cultivation. Then, the resulting monolayers were rinsed twice with PBS, after which the cells were infected with the virus or the antioxidant treatment was administered (2 mM NAC) at an MOI of one before incubation for 0, 1, 2, 3, 4, and 5 days. At different time points after infection, the cells were washed, fixed, and permeabilized with PBS containing 0.2% Triton X-100-PBS for 5 min on ice. Then, they were incubated with 0.05 mM MDC in PBS at 37 °C for 10 min [28]. After incubation, cells were washed four times with PBS and collected in 10 mM Tris-HCl with a pH of 8 and containing 0.1% Triton X-100. Intracellular MDC was measured using fluorescence photometry (with an excitation wavelength of 380 nm and an emission filter of 525 nm) and a Packard Fluorocount microplate reader. To normalize the measurements to the number of cells present in each well, a solution of ethidium bromide was added to a final concentration of 0.2 μM, and the DNA fluorescence was measured (excitation wavelength 530 nm, emission filter 590 nm). The MDC incorporated was expressed as specific activity (arbitrary units).

### 2.5. Transmission Electron Microscopy (TEM)

Trypsinized cells were collected using centrifugation, washed twice with PBS, and then fixed in 2.5% glutaraldehyde in a 0.1 M sodium cacodylate buffer (pH 7.2) for 2 h. The cells were then washed with a sodium cacodylate buffer, post-fixed in 1% aqueous osmium tetroxide for 2 h, and then washed again in the same buffer. They were then dehydrated in a series of ethanol solutions with increasing concentrations and embedded in Spurr’s mixture (14,300, EMS, Hatfield, PA, USA). Semi-thin sections were stained with toluidine blue to facilitate the counting of visualized morphological patterns via light microscopy (Nikon, Inc., Tokyo, Japan). Ultra-thin sections were stained with lead citrate and uranyl acetate and observed using TEM (H-7000, Hitachi Ltd., Tokyo, Japan) [29].

### 2.6. Western Blot Analysis

The GF-1 cells were cultured to monolayer confluence for 20 h in 60 mm Petri dishes (10^5^/mL), rinsed twice with PBS, treated with 2 mM of NAC for 2 h, and then infected with ISKNV (MOI = 1) for 0, 1, 3, and 5 days at 28 °C. At the end of each incubation period, the culture medium was aspirated and the cells were washed with PBS and lysed in 0.3 mL of lysis buffer (comprising 10 mM of Tris, pH 7.3, 20% glycerol, 10 mM of SDS, and 2% ß-mercaptoethanol, pH 6.8). The lysates were separated using sodium dodecyl sulfate–polyacrylamide gel electrophoresis [30], and the proteins were transferred to nitrocellulose. The blots were incubated with (self-made) polyclonal antibodies against MCP, LC3B (GTX127375, GeneTex, San Antonio, TX, USA), MTOR (2972, Cell Signaling Technology, Inc., Danvers, MA, USA), phospho-mTOR (Ser 2448) (2971, Cell Signaling Technology, Inc., Danvers, MA, USA), AMPK (H1213, Santa Cruz, Dallas, TK, USA), phosphor-AMPK (Thr 172) (Bo314, Santa Cruz Dallas, TK, USA), Nrf2 (011356, Enzo Life Sciences, Farmingdale, NY, USA), CAT (sc-27103, Santa Cruz, Dallas, TK, USA), Cu/SOD (206504, CAYMAN CHEMICAL, Ann Arbor, MI, USA), Mn/SOD (TX 116093, GeneTex, San Antonio, TX, USA) and ACTB/ß-actin, followed by peroxidase-labeled goat antirabbit conjugate (1:7500) (MAB1501, Calbiochem, San Diego, CA, USA). Binding was detected using chemiluminescence, and the signals were recorded on Kodak XAR-5 film (Eastman Kodak, Rochester, NY, USA) [31]. Protein expression was quantified using a personal densitometer (Molecular Dynamics, Caesarea, Israel).

### 2.7. Statistical Analysis

Qualitative data, including immunoblots and images, were representative of at least three experiments. The quantification of these results was based on three individual experiments. The results are expressed as the mean ± standard error of the mean. The data were analyzed using a paired or unpaired Student’s *t*-test or an ANOVA with multiple comparisons, as appropriate. A *p*-value below 0.05 (*) or 0.01 (**) was considered statistically significant. SAS version 12.0 was used for all analyses (SAS, Inc., Cary, NC, USA) [32]. 

## 3. Results

### 3.1. Blockage of Oxidative Stress Can Enhance Host Cell Survival and Reduce the Viral Titer of ISKNV Infection in GF-1 Cells

**Figure 1:**
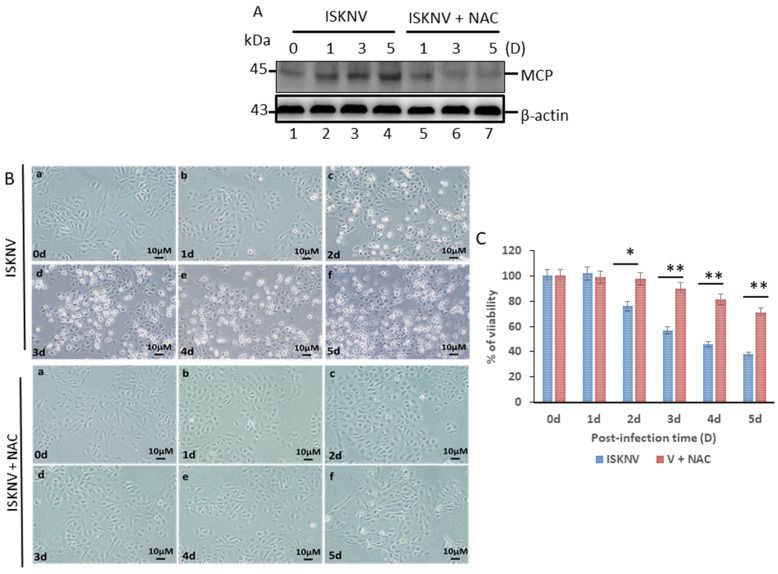
**Identification of ISKNV-induced cell death blocked by antioxidant NAC fish GF-1 cells.** (**A**) Western blot analysis of ISKNV major capsid protein expression in fish cells following infection and incubation for day 0 (lane 1), day 1 (lane 2), day 3 (lane 3), and day 5 (lane 4) and antioxidant NAC (2 mM) treatment for day 1 (lane 5), day 3 (lane 6), and day 5 (lane 7). MCP proteins were detected by Western blot analysis; the gels were immunoblotted with a polyclonal antibody to ISKNV major capsid protein featuring ß-actin as an internal control. (**B**) Phase-contrast micrographs of ISKNV-infected fish cell (a–f) with antioxidant NAC (2 mM) treatment (a–f) from day 0 to day 5, respectively. Phase-contrast images of rounded-up cells were stained with trypan blue to examine cell damage. Scale bar = 10 µm. (**C**) Percentage of cell mortality of ISKNV-infected cells from day 0 to day 5; results were derived from three individual experiments. All data were analyzed using either paired or unpaired Student’s *t*-tests, as appropriate. * *p* < 0.05 and ** *p* < 0.01.

We found that ISKNV viral MCP expression was identified and analyzed using Western blot analysis (Figure 1A); expression of the capsid protein (MCP) was found to have increased by 1.2-, 1.4-, and 2.0-fold by day 1, day 3, and day 5, respectively, which was apparently prevented by antioxidant NAC treatment. Then, antioxidant NAC treatment prevented cell damage (Figure 1B) and enhanced cell viability by up to 21% and 36% after 2 days and 5 days (Figure 1C), respectively. 

### 3.2. ISKNV Infection Induces ROS-Mediated Oxidative Stress Signals within Anti-Oxidative Enzyme Expression in GF-1 Cells

**Figure 2:**
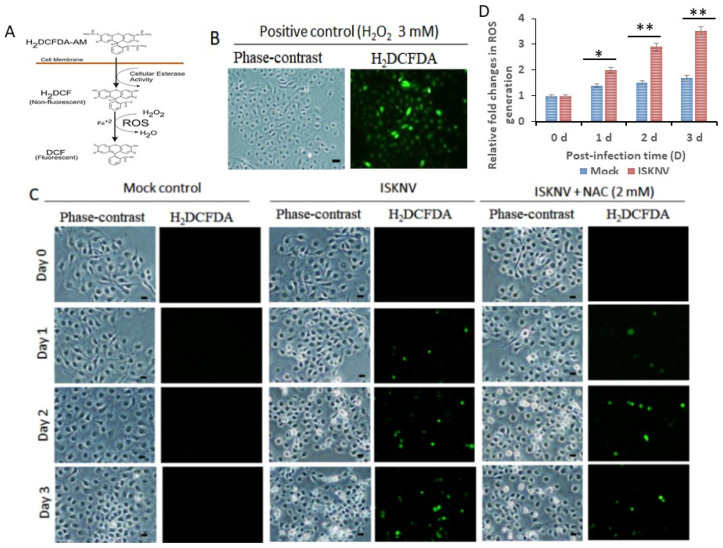
**Blockage of ISKNV induces the production of ROS by antioxidant enzymes in fish GF-1 cells.** (**A**) Cellular esterase activity can conserve H_2_DCFDA in DCF, meaning a shift from non-fluorescence to fluorescence. (**B**) Cells exhibiting ROS generation were treated with H_2_O_2_ (3 mM) for 24 h, causing the green fluorescence indicated by arrows herein. Scale bar = 10 µm. (**C**) Phase-contrast/green fluorescence micrographs of ISKNV-infected and NAC (2 mM)-treated fish cells from 0 to day 3. Phase-contrast images of rounded-up cells stained with H_2_DCFDA to examine ROS production; positive cells are indicated by white arrows. Scale bar = 10 µm. (**D**) Relative fold changes in ROS production (according to the H_2_DCFD assay) by ISKNV-infected cells at different time points. Day 0 of the mock group (i.e., the normal control) represents a 1-fold change in fish cells. The numbers of virus-infected cells in each of the three images are the result of three individual experiments conducted using Image J software (1.50i). All data were analyzed using either paired or unpaired Student’s *t*-tests, as appropriate. * *p* < 0.05 and ** *p* < 0.01.

ISKNV was shown to induce the production of H_2_DCFDA-Am, which then cleaved to H_2_DCFDA and DCF through cellular esterase activity (Figure 2A); this we observed in the positive control (H_2_O_2_ treatment) using green fluorescence cells (3 mM for two hours) (Figure 2B). 

In evaluating ISKNV infection at different time points, we found that ISKNV can quickly induce the production of ROS on day 1 (as indicated by the green color in Figure 2C), then gradually prompt the creation of ROS-positive cells on days 2 and 3 (particularly when compared with mock control groups from day 1 to day 3). More interestingly, we found that antioxidant NAC (2 mM) treatment reduced the presence of ROS-positive cells with ISKNV infection (Figure 2C). Subsequently, our ROS generation assay (Figure 2D) demonstrated that NAC treatment decreased the generation of ROS by up to 0.5-fold (day 1), 1.4-fold (day 2), and 1.8-fold (day 3) compared with the ISKNV-infected group; ISKNV infection proved able to induce the production of ROS in the early stages of replication and was then blocked by antioxidants in GF-1 cells. 

### 3.3. What Host Antioxidant Responses Does Oxidative Stress Provoke?

**Figure 3:**
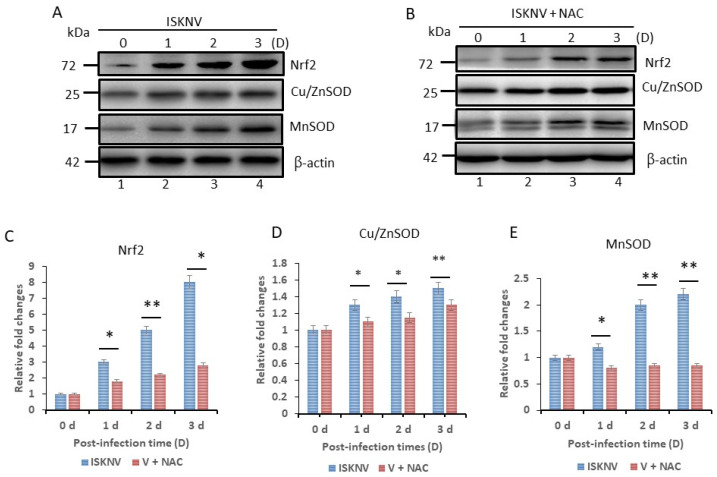
**Identification of ISKNV infection upregulates stress-mediated Nrf2 and antioxidant enzyme expression in GF-1 cells.** (**A**) ISKNV-infected fish cells from day 0 to day 3 and (**B**) NAC-treated and virus-infected cells; also shown are levels of transcriptional factor Nrf2 and antioxidant enzymes Cu/MnSOD (SOD1) and ZnSOD (SOD2), as determined by Western blot analyses. Lanes 1–4 correspond to ISKNV-infected fish cells from day 0 to day 3 post infection, with antibody (1:12,500). (**A**–**E**) show the quantification of protein expression levels and Nrf2 (**C**), Cu/MnSOD (**D**,**E**) ZnSOD (All N = 3) using Image J software (1.50i). All data were analyzed using either paired or unpaired Student’s *t*-tests, as appropriate. * *p* < 0.05 and ** *p* < 0.01.

We found that ISKNV-infected oxidative stress upregulated the antioxidative stress transcriptional factor Nrf2 and the antioxidant enzymes Cu/ZnSOD and MnSOD (Figure 3A) from day 1 to day 3, and that oxidative stress response (via gene upregulation) was repressed by antioxidant NAC treatment (Figure 3B). We then undertook quantification of protein expression levels, as shown in Figure 3C–E, and found that antioxidant NAC treatment caused 1-fold (day 1), 2.5-fold (day 2) and 5-fold decreases (day 3) in Nrf2 (Figure 3C); 0.2-fold changes in Cu/ZnSOD from day 1 to day 3 (Figure 3D); and 0.4-fold (day 1), 1.2-fold (day 2), and 1.4-fold changes (day 3) in MnSOD (Figure 3E), respectively. 

### 3.4. ROS Stress Signaling Is Linked to Autophagy Induction via Puntafomation (MDC)

**Figure 4:**
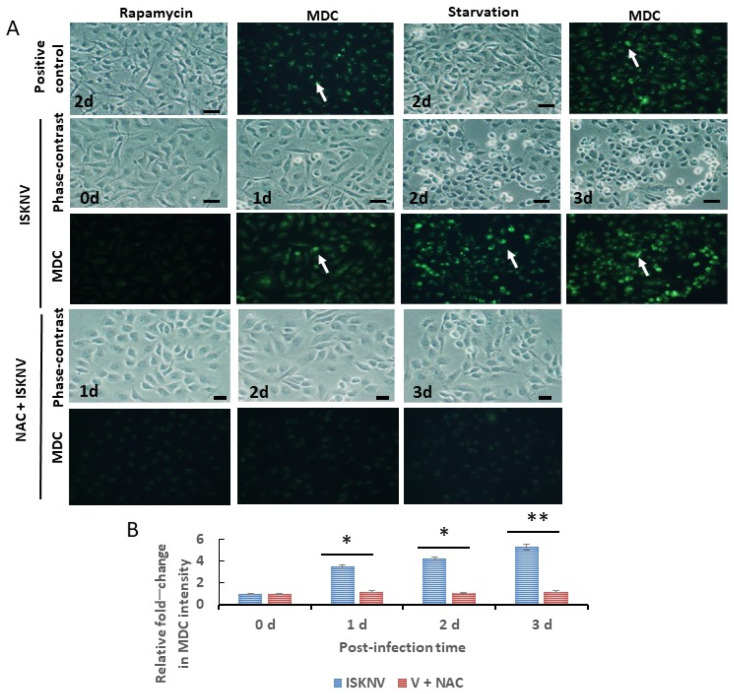
Identification of autophagosome formation through the use of the autofluorescent compound monodansylcadaverine (MDC) for in vitro labeling of autophagic vacuoles in GF-1 cells. (**A**) Autophagosome assay via MDC staining. In the positive control treated with Rapamycin (2 days with a dose of 2 mM) and starvation (2 days of serum withdrawal), positive cells emerged, as indicated by arrows. In the ISKNV-infected (from day 0 to day 3) and NAC-treated groups (from day 1 to day 3), positive cells also emerged, as indicated by arrows. Scale bar = 10 µm. (**B**) Quantification of fold-changes in green fluorescence between ISKNV-infected and NAC-treated groups (all n = 3) using Image J software, as shown in (**A**). V + NAC as ISKNV infected plus NAC treated in GF-1 cells. All data were analyzed using either paired or unpaired Student’s *t*-tests, as appropriate. * *p* < 0.05 and ** *p* < 0.01.

We examined the use of the autofluorescent compound monodansylcadaverine (MDC) for in vitro labeling of autophagic vacuoles in GF-1 cells. Cells subjected to Rapamycin and serum starvation were used as a positive control. Autophagosome formation was observed after 48 h in Rapamycin-treated and serum-starved cells, as indicated by green fluorescence (Figure 4A).

Compared with control cells (0 h), in the ISKNV-infected cells (Figure 4A), autophagosome formation significantly increased in a time-dependent manner, with a ~2.6-fold increase on day 1, a ~3-fold increase on day 2, and a ~4.2-fold increase on day 3 (Figure 4B). Antioxidant NAC treatment appeared to reduce the magnitude of the change in MDC intensity to just one-fold from day 1 to day 3 (Figure 4B).

### 3.5. ROS Stress Signaling Is Linked to Autophagy Induction via Autophagic Markers LC3-II and TEM Criteria 

**Figure 5:**
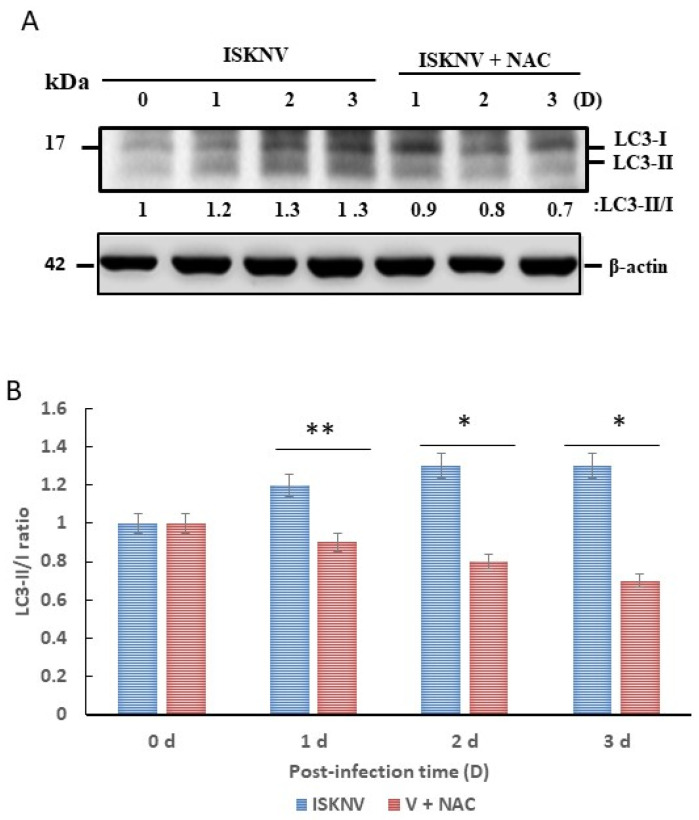
**ISKNV induction of autophagy marker LC3-I conserved to LC3-II by lipidation in GF-1 cells.** (**A**) Western blot analysis of ISKNV infection-induced conversion of LC3-I to LC3-II, as illustrated by ISKNV-infected and NAC-treated groups from day 0 to day 3. LC3-II formation in fish cells following infection, NAC treatment, and incubation after 0 (lane 1), 1 (lane 2), 2 (lane 3), and 3 (lane 4) days with ISKNV; also shown is day 1 (lane 5), day 2 (lane 6), and day 3 (lane 7) in the NAC-treated group. LC3-I and II proteins were detected using Western blot analysis; gels were immunoblotted with a polyclonal antibody to LC3B and ß-actin as an internal control. (**B**) Quantification of LC3-II/I ratio (N = 3) using Image J software, as shown in (**A**). V + NAC as ISKNV infected plus NAC treated in GF-1 cells. All data were analyzed using either paired or unpaired Student’s *t*-tests, as appropriate. * *p* < 0.05 and ** *p* < 0.01.

The expression of proteins increased in the ISKNV-infected cells, including an early marker (LC3B-II). The ratio of the expression of the early autophagy marker LC3-II to LC3-I (LC3-II–LC3-I based on protein fold change) increased in a time-dependent manner from day 0 to day 5 in ISKNV-infected cells (Figure 5A) but was reduced by antioxidant NAC treatment by 0.3-fold (day 1), 0.5-fold (day 2), and 0.6-fold (day 3) (Figure 5B).

**Figure 6:**
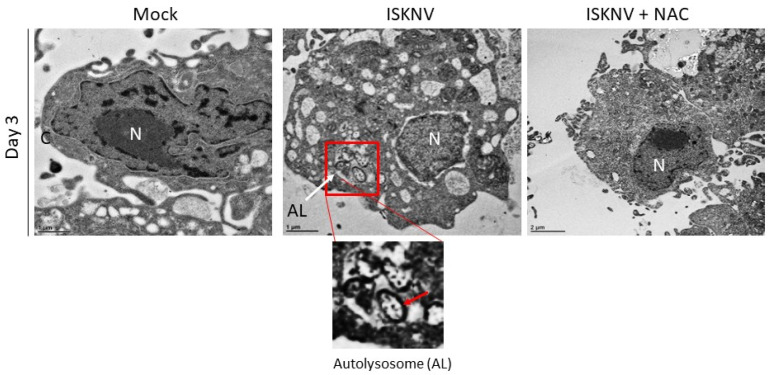
Identification of autophagosomes via transmission electron microscopy at 72 h in the normal control GF-1 cells, ISKNV-infected cells, and NAC plus ISKNV-infected cells. The enlarged images show the autolysosome (AL) and include the damaged mitochondria (M), as indicated by arrows. N: nucleus.

Autophagosome formation was also detected using transmission electron microscopy (TEM) (Figure 6). Damaged mitochondria were found in autolysosomes on day 3 post-ISKNV infection, and the morphology of the uninfected control cells appeared normal; antioxidant NAC appeared to block ISKNV infection, in accordance with the results of the MDC system.

### 3.6. ROS/Nrf2-Mediated Stress Primes AMPK/mTOR Autophagic Signaling to Regulate Viral Replication in GF-1 Cells

**Figure 7:**
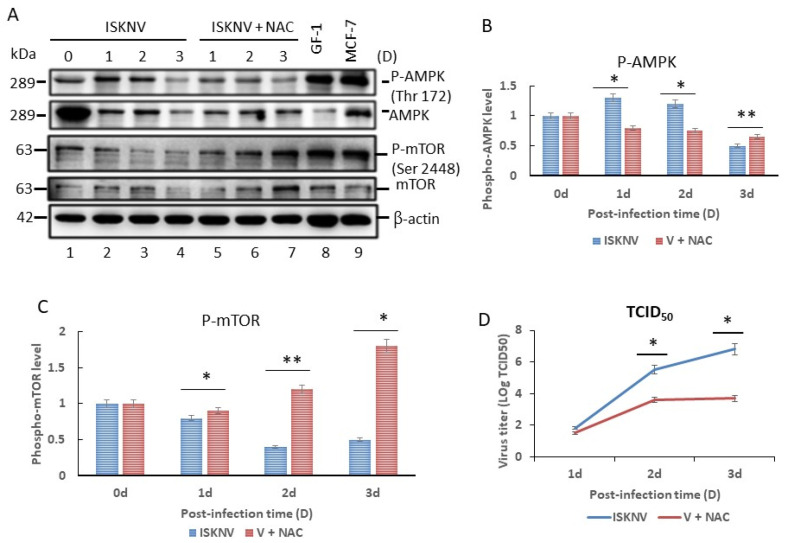
ISKNV infection upregulates mechanistic targets of AMPK and mTOR-mediated autophagy in grouper fin-1 (GF-1) cells, which is blocked by antioxidant NAC. (**A**) Results of our immunoblotting analysis of the expression of AMPK and its downstream molecule MTOR and their phosphorylation following different treatments on day 0, 1, 2, and 3 (lanes 1–4 show the ISKNV-infected group; lanes 5–7 show the NAC + ISKNV group; lane 8 shows the GF-1 cell lysate; lane 9 shows the MCF-7 cell lysate). Quantitation of (**B**) AMPK Thr172 and (**C**) MTOR Ser2448 phosphorylation, as shown in (**A**) (the data were analyzed using an ANOVA with multiple comparisons, where values of * *p* < 0.05 and ** *p* < 0.01 are considered to indicate statistically significant differences between the mean group values). (**D**) Quantification of ISKNV viral titer and the NAC-treated group via a TCID_50_ assay (N = 3), as shown in (**A**). All data were analyzed using either paired or unpaired Student’s *t*-tests, as appropriate. * *p* < 0.05.

We investigated whether H_2_O_2_ signaling is involved in the initiation of autophagy by evaluating the effects of antioxidant NAC on the activation of AMPK and its downstream mediator mTOR (Figure 7A). ISKNV infection increased the phosphorylation level of AMPK Thr172 on day 0 (by 1.0-fold), day 1 (by 1.3-fold), day 2 (by 1.2-fold) and day 3 (by 0.5-fold) after infection (which is the early–middle replication stage). We compared these figures with the NAC-treated group on day 0 (1.0-fold), day 1 (0.8-fold), day 2 (0.75-fold) and day 3 (0.65-fold) (Figure 7B). Furthermore, we identified a reduction in the level of mTOR Ser2448 phosphorylation on day 1 (0.8-fold), day 2 (0.4-fold), and day 3 (0.5-fold) (Figure 7C), serving to enhance the process of autophagy, which was blocked by NAC treatment. mTOR activation is blocked through a spike in the phosphorylation of mTOR Serine 2448. We received consistent results, illustrating that inducing ROS/Nrf2-mediated stress can regulate the process of autophagy within the replication cycle of ISKNV infection.

Additionally, the viral titers (as determined by the TCID_50_ assay) were ~200–360 times lower in NAC-treated cells on days 2 and 3 (*p* < 0.01) (Figure 7D), which indicates that ISKNV-induced ROS-mediated stress signals enhance ISKNV replication in GF-1 cells in the middle–later stages.

## 4. Discussion

In Asia, iridivirus, especially megalocytivirus, has caused severe economic losses (particularly the ISKNV strain) in a worldwide range of economically important marine fish and freshwater species [1,2,3]. In this study, we observed that ISKNV induces host autophagic flux through ROS-mediated stress signaling from the early replication stage (day 1); such signaling is activated by the anti-phosphorylation of AMPK, which suppresses mTOR activation from the early to the middle replication stage (from day 1 to day 3) in fish cells. Finally, the ROS/Nrf2-mediated signal can regulate autophagic flux, which is linked to viral replication. Thus, we conclude that this new discovery of ISKNV-induced autophagy defines a new molecular mechanism of DNA viral pathogenesis and control. 

### 4.1. Oxidative Stress as a Risk Factor in DNA Viruses 

A new aspect of stress signaling by ROS is a phenomenon caused by an over imbalance during the production and accumulation of the ROS in cells or tissues; this further affects the biological system towards a redox reaction with reactive products such as amino acids or lipids. Recently, ROS-mediated stress can play an important role in cell signaling, gene transcription and cell death induction that also strongly correlates with a significant increase in the generation of ROS [33]. Furthermore, some changes in the condition via cell and tissue damage, which induce the imbalance of ROS production, can lead to brain diseases such as Alzheimer’s and Parkinson’s [34,35]. 

Some RNA viruses [36,37,38], DNA viruses [39], and retroviruses [40] can trigger oxidative stress and induce host cell death in infected cells. ISKNV-induced ROS production and its connection to apoptotic pathogenesis has previously been examined [8], but few studies have linked this facet to autophagy; doing so will provide new insights into the treatment and control of these viruses.

In the present study, we found that antioxidant NAC can prevent MCP expression and enhance host cellular viability (Figure 1). We found that ISKNV can prompt the production of ROS (Figure 2), which then triggers ROS/Nrf2-mediated signals (Figure 3) to enhance antioxidant enzyme expression; help the host to regulate their metabolism when alleviating ROS-induced damage; or cause the emergence of autophagy markers such as LC3-II (Figure 5), the formation of autophagosomes (Figure 6), and AMPK/mTOR signaling flux (Figure 7) in association with viral replication (Figure 7D).

### 4.2. The Effect of AMPK/mTOR Signaling on Infection with DNA Viruses 

Recently, research has very quickly accumulated results on cellular autophagy flux. This process is induced by some factors or conditions such as nutrient starvation; cells are primed through an autophagosome induction and a lysosomal-dependent self-digestive process. Through this process, cells can digest cytoplasmic contents, such as damaged proteins and organelles, hydrolyzing them to generate nutrients and energy for maintaining essential cellular activities [41,42,43,44]. Autophagy flux is a tightly regulated process, and blockage of autophagy is strongly associated with many human diseases including cancer, heart, and neurodegeneration [45,46,47]. Moreover, the clearance of pathogens and antigen presentation are involved in the autophagy process [48,49,50].

The mTOR is a serine/threonine kinas that has been identified as an evolutionarily conserved functional domain which can act as a central negative regulator of autophagy in the early initiation stage. Up to now, some factors can regulate this molecule such as starvation, oxidative stress, energy stress, and pathogen infection by phosphorylation for the inhibition of mTOR. The upstream signaling is controlled by either the protein kinase B/ (PKB/AKT) pathway or tuberous sclerosis complex 2 (TSC2) to activate mTOR complex 1 (mTORC1), subsequently inhibiting autophagy [51,52]. On the other hand, AMPK, as a positive regulator on autophagy induction, can play a key role in sensing cellular energy and ATP levels [53,54]. In the host cell induced by DNA viruses, few can regulate the AMPK/mTOR signaling pathway. We found that ISKNV infection can trigger AMPK/mTOR autophagy flux, as shown in Figure 7; this result is supported by enteritis virus (DEV) infection that both activates the metabolic regulator AMPK and inhibits mTOR activity [55]. 

### 4.3. The Effect of Autophagy Flux on DNA Viral Replication 

Autophagy flux can dominate the effect of cell homeostasis during viral pathogen infection in DNA virus infection, which also correlates with the cause of disorders. Recently, virus infections can interact with the host cell, which triggers this process. Autophagy can regulate innate immunity by inflammasome formation on IL-1β activation to produce an antiviral effect. In contrast, viruses often express their gene products to overcome and resist this process. From an evolutionary perspective, a virus can benefit itself by hijacking the autophagy process to enhance its own replication at the latent infection stage [56]. Some cases have shown either pro- or antiviral effects of autophagy during infection with DNA viruses of great interest, such as Epstein–Barr virus (EBV) [57] and porcine circovirus type 2 (PCV2) [58]. In our study, we observed that ISKNV infection can induce the process of autophagy via puntaformation (Figure 4) and LC3-II (Figure 5A) modification, which further activated AMPK/mTOR autophagy signaling to enhance viral replication, especially after the middle stages of the replication cycle (day 3); the reasons for this chain of events remain unclear.

## 5. Conclusions

**Figure 8:**
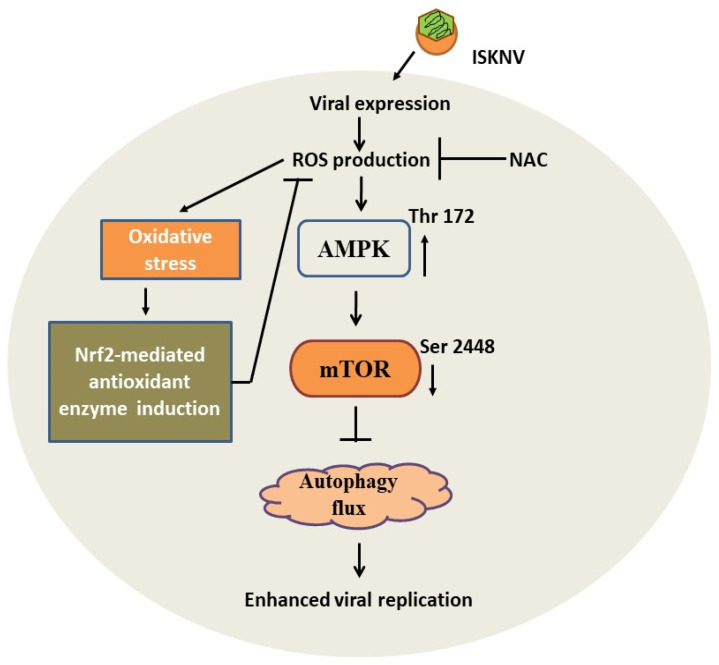
The hypothesis that ISKNV triggered ROS-mediated stress signals both modulate the autophagic flux pathway and anti-oxidants response on enhancement of viral replication in fish cells. The ISKNV shows a novel approach, using ROS-mediated stress response that can either activate the AMPK/mTOR autophagy signaling pathway via phosphorylation or induce the oxidative defense stress signal in the early replication stages in fish cells. The ISKNV-induced AMPK/mTOR autophagy signaling pathway is primed by ROS-mediated stress signals on either phosphorylation of AMPK on threonine 172 or suppresses phosphorylation of mTOR on serine 1448 for autophagy induction. On the other hand, the oxidative stress response also upregulates the stress transcriptional factor Nrf2 and antioxidant enzymes such as Cu/MnSOD (SOD1) and ZnSOD (SOD2), which are correlated with metabolized ROS, such as superoxide, for the recovery of biological functions in proteins. Thus, ISKNV induced ROS-mediated AMPK/mTOR autophagy pathway on enhanced viral replication that can be inhibited by treatment with antioxidants NAC, which provides a novel strategy for viral control.

Taken together, our results suggest that a novel ISKNV infection can trigger ROS/Nrf2-related stress signals for the subsequent regulation and induction of host autophagy (Figure 8). Said stress signals are both strongly correlated with the upregulation of the antioxidant enzymes Cu/ZnSOD and MnSOD, which serve to overcome ROS-mediated stress and trigger autophagic flux via the AMPK/mTOR signaling pathway as well as enhance viral replication in the middle–late replication cycle. Finally, we found that NAC treatment blocked ISKNV-triggered ROS/Nrf2 stress signals and either suppressed the AMPK/mTOR-related autophagy pathway or reduced viral replication in fish cells. These findings represent novel insights into DNA viruses and the function of using the host-stress response to initiate a dynamic control strategy through which to regulate antioxidant response, autophagy flux, and viral replication.

## Data Availability

The datasets used and/or analyzed in the current study are available from the corresponding author upon reasonable request.
